# Evidence of causality of low body mass index on risk of adolescent idiopathic scoliosis: a Mendelian randomization study

**DOI:** 10.3389/fendo.2023.1089414

**Published:** 2023-06-20

**Authors:** Nao Otomo, Anas M. Khanshour, Masaru Koido, Kazuki Takeda, Yukihide Momozawa, Michiaki Kubo, Yoichiro Kamatani, John A. Herring, Yoji Ogura, Yohei Takahashi, Shohei Minami, Koki Uno, Noriaki Kawakami, Manabu Ito, Tatsuya Sato, Kei Watanabe, Takashi Kaito, Haruhisa Yanagida, Hiroshi Taneichi, Katsumi Harimaya, Yuki Taniguchi, Hideki Shigematsu, Takahiro Iida, Satoru Demura, Ryo Sugawara, Nobuyuki Fujita, Mitsuru Yagi, Eijiro Okada, Naobumi Hosogane, Katsuki Kono, Masaya Nakamura, Kazuhiro Chiba, Toshiaki Kotani, Tsuyoshi Sakuma, Tsutomu Akazawa, Teppei Suzuki, Kotaro Nishida, Kenichiro Kakutani, Taichi Tsuji, Hideki Sudo, Akira Iwata, Satoshi Inami, Carol A. Wise, Yuta Kochi, Morio Matsumoto, Shiro Ikegawa, Kota Watanabe, Chikashi Terao

**Affiliations:** ^1^ Laboratory for Statistical and Translational Genetics, RIKEN Center for Integrative Medical Sciences, RIKEN, Yokohama, Japan; ^2^ Department of Orthopaedic Surgery, Keio University School of Medicine, Tokyo, Japan; ^3^ Laboratory for Bone and Joint Diseases, RIKEN Center for Integrative Medical Sciences, Tokyo, Japan; ^4^ Center for Translational Research, Scottish Rite for Children, Dallas, TX, United States; ^5^ Division of Molecular Pathology, Institute of Medical Science, The University of Tokyo, Tokyo, Japan; ^6^ Laboratory for Genotyping Development, RIKEN Center for Integrative Medical Sciences, Yokohama, Japan; ^7^ Laboratory of Complex Trait Genomics, Graduate School of Frontier Science, The University of Tokyo, Tokyo, Japan; ^8^ Department of Orthopaedic Surgery , Scottish Rite for Children, Dallas, TX, United States; ^9^ Department of Orthopaedic Surgery and Pediatric, University of Texas Southwestern Medical Center, Dallas, TX, United States; ^10^ Department of Orthopaedic Surgery, Seirei Sakura Citizen Hospital, Sakura, Japan; ^11^ Department of Orthopaedic Surgery, National Hospital Organization, Kobe Medical Center, Kobe, Japan; ^12^ Department of Orthopaedic Surgery, Meijo Hospital, Nagoya, Japan; ^13^ Department of Orthopaedic Surgery, National Hospital Organization, Hokkaido Medical Center, Sapporo, Japan; ^14^ Department of Orthopaedic Surgery, Juntendo University School of Medicine, Tokyo, Japan; ^15^ Department of Orthopaedic Surgery, Niigata University Medical and Dental General Hospital, Niigata, Japan; ^16^ Department of Orthopaedic Surgery, Osaka University Graduate School of Medicine, Suita, Japan; ^17^ Department of Orthopaedic and Spine Surgery, Fukuoka Children’s Hospital, Fukuoka, Japan; ^18^ Department of Orthopaedic Surgery, Dokkyo Medical University School of Medicine, Mibu, Japan; ^19^ Department of Orthopaedic Surgery, Kyushu University Beppu Hospital, Beppu, Japan; ^20^ Department of Orthopaedic Surgery, Faculty of Medicine, The University of Tokyo, Tokyo, Japan; ^21^ Department of Orthopaedic Surgery, Nara Medical University, Kashihara, Japan; ^22^ Department of Orthopaedic Surgery, Dokkyo Medical University Saitama Medical Center, Koshigaya, Japan; ^23^ Department of Orthopaedic Surgery, Teine Keijinkai Hospital, Sapporo, Japan; ^24^ Department of Orthopaedic Surgery Graduated School of Medical Science, Kanazawa University, Kanazawa, Japan; ^25^ Department of Orthopaedic Surgery, Jichi Medical University, Shimotsuke, Japan; ^26^ Department of Orthopaedic Surgery, Fujita Health University, Toyoake, Japan; ^27^ Department of Orthopaedic Surgery, International University of Health and Welfare School of Medicine, Narita, Japan; ^28^ Department of Orthopaedic Surgery, National Defense Medical College, Tokorozawa, Japan; ^29^ Department of Orthopaedic Surgery, Kono Orthopaedic Clinic, Tokyo, Japan; ^30^ Department of Orthopaedic Surgery, Kobe University Graduate School of Medicine, Kobe, Japan; ^31^ Department of Advanced Medicine for Spine and Spinal Cord Disorders, Hokkaido University Graduate School of Medicine, Sapporo, Japan; ^32^ Department of Preventive and Therapeutic Research for Metastatic Bone Tumor, Faculty of Medicine and Graduate School of Medicine, Hokkaido University, Sapporo, Japan; ^33^ McDermott Center for Human Growth and Development, University of Texas Southwestern Medical Center, Dallas, TX, United States; ^34^ Department of Genomic Function and Diversity, Medical Research Institute, Tokyo Medical and Dental and University, Tokyo, Japan; ^35^ Clinical Research Center, Shizuoka General Hospital, Shizuoka, Japan; ^36^ Department of Applied Genetics, The School of Pharmaceutical Sciences, University of Shizuoka, Shizuoka, Japan

**Keywords:** adolescent idiopathic scoliosis, body mass index, Mendelian randomization (MR), genetic study, genome-wide association study

## Abstract

**Introduction:**

Adolescent idiopathic scoliosis (AIS) is a disorder with a three-dimensional spinal deformity and is a common disease affecting 1-5% of adolescents. AIS is also known as a complex disease involved in environmental and genetic factors. A relation between AIS and body mass index (BMI) has been epidemiologically and genetically suggested. However, the causal relationship between AIS and BMI remains to be elucidated.

**Material and methods:**

Mendelian randomization (MR) analysis was performed using summary statistics from genome-wide association studies (GWASs) of AIS (Japanese cohort, 5,327 cases, 73,884 controls; US cohort: 1,468 cases, 20,158 controls) and BMI (Biobank Japan: 173430 individual; meta-analysis of genetic investigation of anthropometric traits and UK Biobank: 806334 individuals; European Children cohort: 39620 individuals; Population Architecture using Genomics and Epidemiology: 49335 individuals). In MR analyses evaluating the effect of BMI on AIS, the association between BMI and AIS summary statistics was evaluated using the inverse-variance weighted (IVW) method, weighted median method, and Egger regression (MR-Egger) methods in Japanese.

**Results:**

Significant causality of genetically decreased BMI on risk of AIS was estimated: IVW method (Estimate (beta) [SE] = -0.56 [0.16], p = 1.8 × 10^-3^), weighted median method (beta = -0.56 [0.18], p = 8.5 × 10^-3^) and MR-Egger method (beta = -1.50 [0.43], p = 4.7 × 10^-3^), respectively. Consistent results were also observed when using the US AIS summary statistic in three MR methods; however, no significant causality was observed when evaluating the effect of AIS on BMI.

**Conclusions:**

Our Mendelian randomization analysis using large studies of AIS and GWAS for BMI summary statistics revealed that genetic variants contributing to low BMI have a causal effect on the onset of AIS. This result was consistent with those of epidemiological studies and would contribute to the early detection of AIS.

## Introduction

### AIS is a problem

Adolescent idiopathic scoliosis (AIS) is a disorder with a three-dimensional spinal deformity. AIS is defined as scoliosis that develops during adolescence with a Cobb angle (CA) of 10 degrees or more on plain radiographs in the standing position (1, 2). AIS is a common disease affecting 1-5% of adolescents (3), progressing rapidly during periods of rapid growth (4), and is associated with physical (5), mental (6), and respiratory problems (7). To prevent these problems, identification of the cause of the onset of AIS is demanded. In Japan, AIS has been adopted as one of the screening factors for adolescents and is considered an important adolescent health problem.

### Etiology of AIS

AIS is also known as a complex disease involved in environmental and genetic factors. In twin’s studies, he concordance of AIS between twins in monozygotic pair is as high as 0.73 to 0.92 and its heritability is estimated as 87.5% (8–11), suggesting that genetic factors account for a large fraction of AIS onset. Research groups including us have identified 20 GWAS significant loci in AIS (12).

### AIS and BMI

BMI is also known as a highly polygenic trait with 670 GWAS significant loci identified so far (13). A relationship between AIS and body mass index (BMI) has been epidemiologically (12) suggested. A clinical study has reported that AIS patients had a significantly lower BMI in comparison with those healthy adolescents (14, 15). We showed a negative genetic correlation between AIS and BMI, which indicates that AIS and BMI share genetic backgrounds or polygenicity with opposing directions of effects (12, 16). This is in line with the epidemiological association between the onset of AIS and low BMI. However, the causal relationship between AIS and BMI remains to be elucidated.

### Mendelian randomization

**Figure 1:**
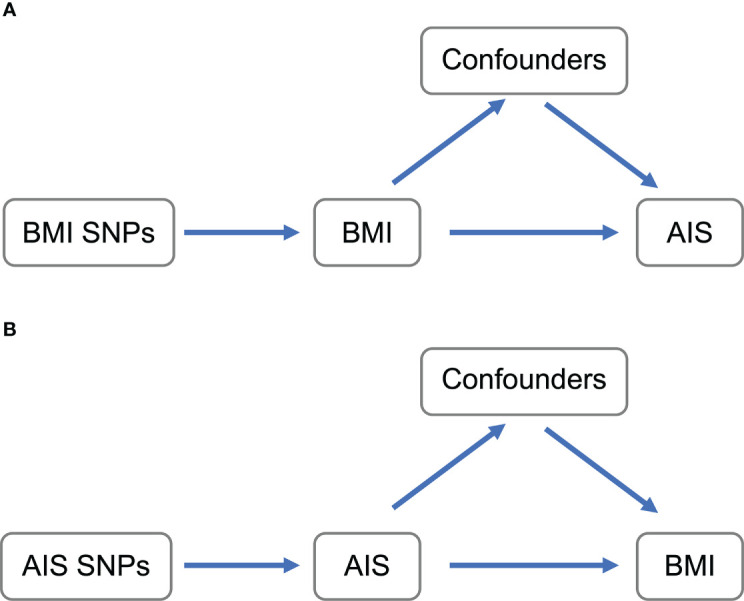
Schematic representation of MR analyses. **(A)** BMI SNPs were used as instrumental variables to investigate the causal effect of BMI upon AIS. **(B)** AIS SNPs were used as instrumental variables to investigate the causal effect of genetic risk of AIS upon BMI. Arrows indicate MR assumption such that the instrumental variable is associated with the exposure—not associated with confounders—and only affects the outcome via the exposure. AIS, adolescent idiopathic scoliosis; BMI, body mass index; MR, mendelian randomization; SNP, single-nucleotide polymorphism.

Using genetic data is becoming popular to resolve those phenotypic relationships (17). Mendelian randomization (MR) is an approach to summarize causal inference between phenotypes using the genome-wide association study (GWAS) results (18, 19). The genetically determined phenotypic profiles are robust for lifelong confounding factors, which can be interpreted as ideal randomization of the subject. Because of the development of MR analytic methods (20) and the achievement of large-scale GWASs of various human phenotypes with publicly available data, MR is one of the best methods to infer causality. Here we conducted MR analysis to investigate the causal inference between BMI and the onset of AIS (**Figure 1**).

## Materials and methods

### Subjects

The ethical committees at all collaborating institutions and RIKEN approved the study. Informed consent was obtained from subjects and/or parents. The details about collecting samples and inclusion criteria have been shown in the previous studies (12, 21–24). In the Japanese AIS cohort, the age of the case (93% of female ratio) was 10 -18 years, and the age of the controls (46% of female ratio) was 18 -100 years. To evaluate the causal relationship in a trans-ethnic manner, we also used another GWAS for AIS summary statistic derived from European ancestry. The age of the case (86% of female ratio) was 12.2 -17 years and the age of the control (55% of female ratio) was 44 -88 years for control.

### GWAS of European ancestry

We used AIS GWAS summary statistics derived from two US cohorts of European ancestry: Missouri (MO1) described in a previous study (25) and Texas (TX). The cases in the TX cohort (n=1,358) were recruited at Texas Scottish Rite Hospital for Children as approved by the Institute Review Board of the University Texas Southwestern Medical Center as previously described (26) and genotyped on the Illumina HumanCoreExome BeadChip. For controls in the TX cohort, we utilized 12,507 non-AMD GRU (non-age related macular degeneration general research use) subjects of European ancestry, downloaded from dbGaP website (https://www.ncbi.nlm.nih.gov/gap) from the International Age-Related Macular Degeneration Genomics Consortium study (IAMDGC: phs001039.v1.p1.c1). Cases and controls genotypes were merged and imputed as described in a previous study (25). Then only bi-allelic common (MAF>0.01) SNPs with imputation quality Rsq >=0.3 were included for further analysis. Genetic association for the imputed allele dosages was performed in Mach2dat (27) using logistic regression with gender and 10 principal components as covariates. The major histocompatibility complex (MHC) region was excluded form analysis.

### Instrumental variables

For AIS, we used published summary statistics of Japanese AIS (12). For summary statistics of European AIS, a new GWAS was performed, the details of which are described above.

For BMI, we used published summary statistics derived from Japanese populations based on the Biobank Japan project (range of 18 -100 years, 46% of female ratio) (16). For European ancestry, we used two published summary statistics derived from European populations; a meta-analysis of GWAS on BMI in UK biobank (range of 40 -69 years, 54% of female ratio) and genetic investigation of anthropometric traits (GIANT) (range of 40 -75 years, 55% of female ratio) (13), and a result of GWAS for BMI of European children (range of 2 -10 years, 51% of female ratio) (28). In addition, summary statistics from the population architecture using genomics and epidemiology (PAGE) consortium (range of 18 -79 years, 50% of female ratio), including African American, Asian, Hispanic, Native American, and Native Hawaiian populations quantities were also used (29). In the above three BMI GWASs, age and sex were added as covariates for the analysis.

The overlapped significant SNPs between BMI and AIS GWAS were used for the analysis (**Table 1**). The Analyses were performed for each population. In the above three BMI GWASs, age and sex were added as covariates for the analysis.

**Table 1:** The characteristics and source of genetic instruments used in MR analyses.

Population of AIS	Instruments form of BMI	Sample size	Number of SNPs		Reference
			Used in MR	Used in reverse MR	
Japanese	Japanese (Biobank Japan)	173,430	70	13	Akiyama et al., 2017 (16)
US	European (UK biobank + GIANT)	806,334	537	NA	Pulit et al., 2019 (13)
	European child	39,620	31	NA	Vogelezang et al., 2020 (28)
	PAGE (Non-European)	49,335	61	NA	Wojcik et al., 2019 (29)

MR, mendelian randomization; AIS, adolescent idiopathic scoliosis; BMI, body mass index; SNP, single nucleotide polymorphism; GIANT, Genetic Investigation of Anthropometric Traits; PAGE, Population Architecture using Genomics and Epidemiology.

Number of SNPs used in MR analysis represent the overlapped BMI associated SNPs between BMI GWAS and AIS GWAS.

Number of SNPs used in reverse MR analysis represent the overlapped AIS associated SNPs between BMI GWAS and AIS GWAS.

NA, not available.

We also evaluated the F-statistics metric to include the strength of genetic instruments for all instruments in different populations. Based on the previous studies, the F-statistic metric was calculated by using the formula: F = (R2/k)/([1 − R2]/[n − k – 1]). R2 represents the proportion of variance explained by the exposure SNP-instruments, k represents the number of instruments, and N represents the exposure GWAS sample size (30, 31).

### Mendelian randomization analysis for the association between AIS and BMI

MR is a method for evaluating the causal effect of a risk factor on an outcome from observational data using genetic variants (32, 33). We adopted two-sample MR which is one of the MR analysis approaches. Two sample MR in which SNP exposure and SNP outcome associations are estimated handling GWAS summary statistics derived from independent studies was applied to the analysis. In this study, we focused on the causality of BMI to AIS using this method implemented in the R software “TwoSampleMR” which is composed of the R language (19). The instruction of this software also provides source codes for analysis. The association between BMI and AIS was evaluated using the inverse-variance weighted (IVW) method, a typical and conventional method in MR, to obtain a weighted average of the effect estimates.

### Sensitivity analysis

If SNPs act through a pleiotropic pathway, it would violate the MR assumption that the instrumental variable affects the outcome only via exposure and bias the causal estimate (that is, the effect of a genetic variant on the outcome acts entirely through the exposure of interest.). Because testing the validity of these assumptions is difficult in indeed, therefore, we conducted additional two MR methods in addition to IVW, namely Egger regression method (MR-Egger) and the weighted median estimator method (Weighted median), to check for the robustness of the estimates for IVW. MR-Egger is similar to IVW other than that the intercept is not limited to passing through the origin, and the nonlinear intercept indicates the possibility of directional pleiotropy (20, 34). MR-Egger is statistically less powerful but can adjust the pleiotropy. The Weighted median method works even when at least half of the subset of genetic variants of the instrumental variables are valid and provides a valid causal estimate (35). The number of SNPs used in each analysis is described in **Table 1**. In Japanese, we also evaluated potential bias in the results of MR analyses by sensitivity analyses including leave-one-out analysis, intercept test for MR-Egger followed in the R software “TwoSampleMR” which also provides source codes for these analyses and global test followed in the R software “MR-PRESSO” (36).

We performed the reverse-direction MR analysis inferring the causality of AIS on BMI. For Japanese overlapped significant SNPs reported in Japanese GWAS for AIS were used as instrumental variables (**Table 2**) (12). Unfortunately, the number of independent SNPs passing the genome-wide significant threshold (p<5×10^-8^) was too small to be analyzed using US AIS summary statistics.

**Table 2:** Results of the MR analyses and the reverse direction MR analyses.

Population of AIS	Instruments form of BMI			Method	MR			Revers MR		
	Cohort	R2	F statistics		Estimate	SE	P-value	Estimate	SE	P-value
Japanese	Japanese (Biobank Japan)	2.80	71.34	IVW	-0.56	0.16	1.1×10^-3^	-0.01	0.03	0.62
				Weighted median	-0.56	0.18	5.1×10^-3^	0.00	0.01	0.61
				MR Egger	-1.50	0.43	2.8×10^-3^	0.02	0.07	0.77
US	European (UK biobank + GIANT)	3.91	60.90	IVW	0.17	0.14	0.22	NA		
				Weighted median	-0.20	0.24	0.42			
				MR Egger	-0.48	0.36	0.18			
	European child	2.92	38.14	IVW	-0.31	0.20	0.12	NA		
				Weighted median	-0.51	0.23	0.03			
				MR Egger	-0.71	0.59	0.24			
	PAGE (Non-European)	7.21	62.67	IVW	-0.79	0.12	9.1×10^-12^	NA		
				Weighted median	-0.91	0.16	1.5×10^-8^			
				MR Egger	-1.74	0.40	4.8×10^-5^			

BMI, body mass index; AIS, adolescent idiopathic scoliosis; MR, mendelian randomization; SE, standard error; GIANT, Genetic Investigation of Anthropometric Traits; PAGE, Population Architecture using Genomics and Epidemiology; R2, variance explained; IVW, inverse variance-weighted; NA, not available.

MR analyses infer causality of the BMI on AIS. Reverse MR analyses infer causality of the AIS on BMI. P value means false discovery rate by Boneferroni’s correction.

### Trans-ethnic genetic correlation

To estimate the genetic correlation of GWAS SNPs from different populations, the python package, popcorn (version 0.9.9; https://github.com/brielin/popcorn), was used (37). East Asian and European data of the 1000 Genomes Project (1KG) were used to compute cross-population scores considering each linkage disequilibrium (LD) structure (38). Consequently, a high genetic correlation of AIS between Japanese and Europeans (rho:0.87 (SE:0.11)) was observed.

### Power calculation for mendelian randomization analysis

We performed power calculations for MR regarding the previous study (39). Using the GWAS data of the Japanese cohort as a reference, we set input parameters including sample size (79,211), the proportion of cases in the study (0.067), type I error rate (0.05), and true odds ratio of the outcome variable per standard deviation of the exposure variable calculated from the estimate of MR analysis with Japanese BMI and AIS GWAS result.

### Polygenic assessment for shared common variants between BMI and AIS

Since MR analysis using summary statistics with different populations is not recommended, we assessed genetic backgrounds between BMI and AIS. The correlation of the odds ratios of the genome-wide SNPs, excluding the BMI and AIS-associated loci, were estimated for sets of SNPs stratified by p-values of GWAS thresholds in each trait. For the shared SNPs between the two traits, LD pruning of the SNPs (r2<0.3) was adopted at first. When stratifying the SNPs based on the p-values in each GWAS, the correlation of odds ratios of the SNPs between BMI and AIS was evaluated by Pearson’s correlation coefficient. This approach has been established to assess the direction of genetic risk between traits or populations as a previous study reported (40).

## Results

### Mendelian randomization analyses of the association between BMI and AIS risk

**Figure 2:**
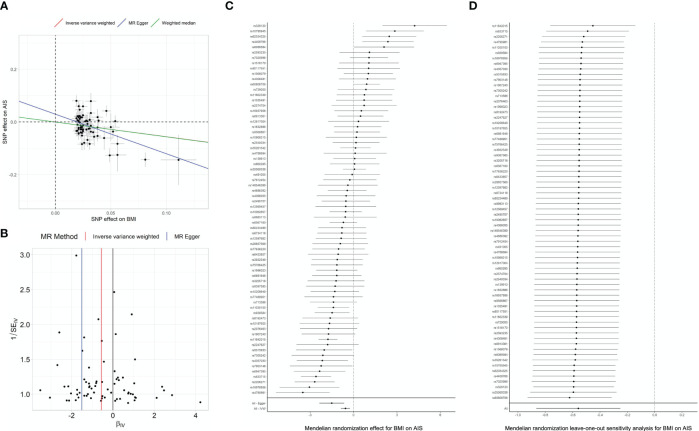
MR analyses and sensitivity analyses in Japanese. **(A)** Regression plot of MR analyses. Dots represent the BMI-associated SNPs plotted along with effect size estimates on BMI (x-axis) and psoriasis risk (y-axis) with 95% confidence intervals in the Japanese population. Regression lines obtained from the MR analyses are plotted in red (by IVW), blue (by MR-Egger) and purple (by Weighted median). BMI, body mass index; IVW, inverse variance weighted; MR, Mendelian randomization; SNP, single nucleotide polymorphism. Sensitivity analysis results evaluating the MR causal relationships of BMI on the risk of Adolescent idiopathic scoliosis. Panels indicate funnel plot **(B)**, heterogeneity test **(C)** and leave-one-out analysis **(D)** in Japanese population.

The primary analyses included 83 SNPs in the Japanese GWAS as instruments for BMI (16). We found significant causality of genetically decreased BMI on the risk of AIS (estimate=-0.56, P=1.110^-3^ in IVW) (**Figure 2**; **Table 2**). The significant causality was also supported in the weighted median method (estimate=-0.56, P=5.110^-3^) and the MR-Egger method (estimate=-1.50, P=22.8×10^-3^) (**Table 2**). The direction of estimates was consistent across all results, indicating that the risk SNPs of increased BMI had suppressive effects on the onset of AIS. These results are consistent with that of an epidemiological study (14). We conducted sensitivity analyses, intercept tests of MR-Egger and MR-PRESSO to evaluate potential bias in the results of MR analysis. According to the MR-Egger intercept test and MR-PRESSO, we did not observe obvious horizontal pleiotropy (**Table 3**).

**Table 3:** Result of MR Egger intercept test and MR-PRESSO for horizontal pleiotropy.

AIS cohort	BMI cohort	MR				Reverse MR			
		MR Egger			MR-PRESSO	MR Egger			MR-PRESSO
		Intercept	SE	P-value	Global test P-value	Intercept	SE	P-value	Global test P-value
Japanese	Japanese (Biobank Japan)	0.03	0.01	0.07	0.52	0.00	0.01	0.58	0.06
US	European (UK biobank + GIANT)	0.01	0.01	0.235	0.12	NA			
	European child	0.02	0.03	0.48	0.08				
	PAGE	0.05	0.02	0.07	0.81				

BMI, body mass index; AIS, adolescent idiopathic scoliosis; MR, mendelian radomization; SE, standard error; GIANT, Genetic Investigation of Anthropometric Traits; PAGE, Population Architecture using Genomics and Epidemiology.

MR analyses infer causality of the BMI on AIS. Reverse MR analyses infer causality of the AIS on BMI.

P value means false discovery rate by Boneferroni’s correction.

Next, we addressed whether the causal inference could be generalizable regardless of the selection of instruments. BMI GWASs have been mainly conducted in European populations. In US GWAS, we observed the analysis was conducted without any problems (λ_GC_=1.03) and three genome-wide significant signals were observed. Since US GWAS has a small sample size, we conducted a trans-ethnic genetic correlation of AIS between Japanese and Europeans using the Popcorn program (37) and observed a high genetic correlation of AIS (rho:0.87 (SE:0.11)). Therefore, to address the generalizability of this inference on AIS and to confirm this trend in different populations, we conducted MR analysis using summary statistics of GWAS for AIS from the US population (European ancestry) and analyzed in three methods (IVW, weighted median method, and MR-Egger) as in the Japanese AIS analysis. We repeated the analyses, using 537 of the 640 BMI-associated SNPs identified by the meta-analysis of the UK biobank and GIANT consortium, 31 of 43 BMI-associated SNPs identified by GWAS for BMI derived from European children, and 61 of the 63 BMI-associated SNPs identified by PAGE consortium (**Table 1**).

Before the analysis, the power calculation was performed to evaluate the results more conservatively. This calculation is based on each variant, not summary statistics. Therefore, we selected two variants, rs11642015 and rs633715, showing the highest association in the Japanese analysis. The power for MR analysis was 0.63 for rs11642015 and 0.36 for rs633715. On the other hand, when we tried to obtain the same results with the US cohort as those of the Japan cohort, the power was 0.23 for rs11642015 and 0.17 for rs633715. There is no significant difference in the case ratios between the Japanese and US cohorts, indicating that the sample size has a pure effect. Additionally, the total sample size was calculated to be 80,918 for rs11642015 and 80,515 for rs633715 to obtain the same power as in Japan with the same cohort ratio. These results indicate that the results of this MR analysis are mainly due to the small sample size of the US cohort.

According to the result of power calculation for MR analysis, a small sample size of the US GWAS lowered statistical power. However, even though most of the results were not significant, but showed consistent trends with those using summary statistics of Japanese GWAS for AIS were observed (**Table 2**). We also repeated the sensitivity analyses, intercept test and MR-PRESSO for each BMI instrument using both of AIS instruments (**Table 3**). We did not find apparent bias by sensitivity analyses, and similar trends like MR analyses were observed between Japanese and US instruments. Finally, we applied the reverse MR analysis inferring the causality of AIS on BMI (**Table 1**). We did not observe evidence of the causality of AIS on BMI in any MR methods (**Table 2**). Since the number of independent SNPs passing genome-wide significant threshold (p<5×10^-8^) were too small (3 SNPs) for reverse MR to be analyzed using US AIS summary statistic.

### Polygenic assessment for shared common variants between BMI and AIS

**Figure 3:**
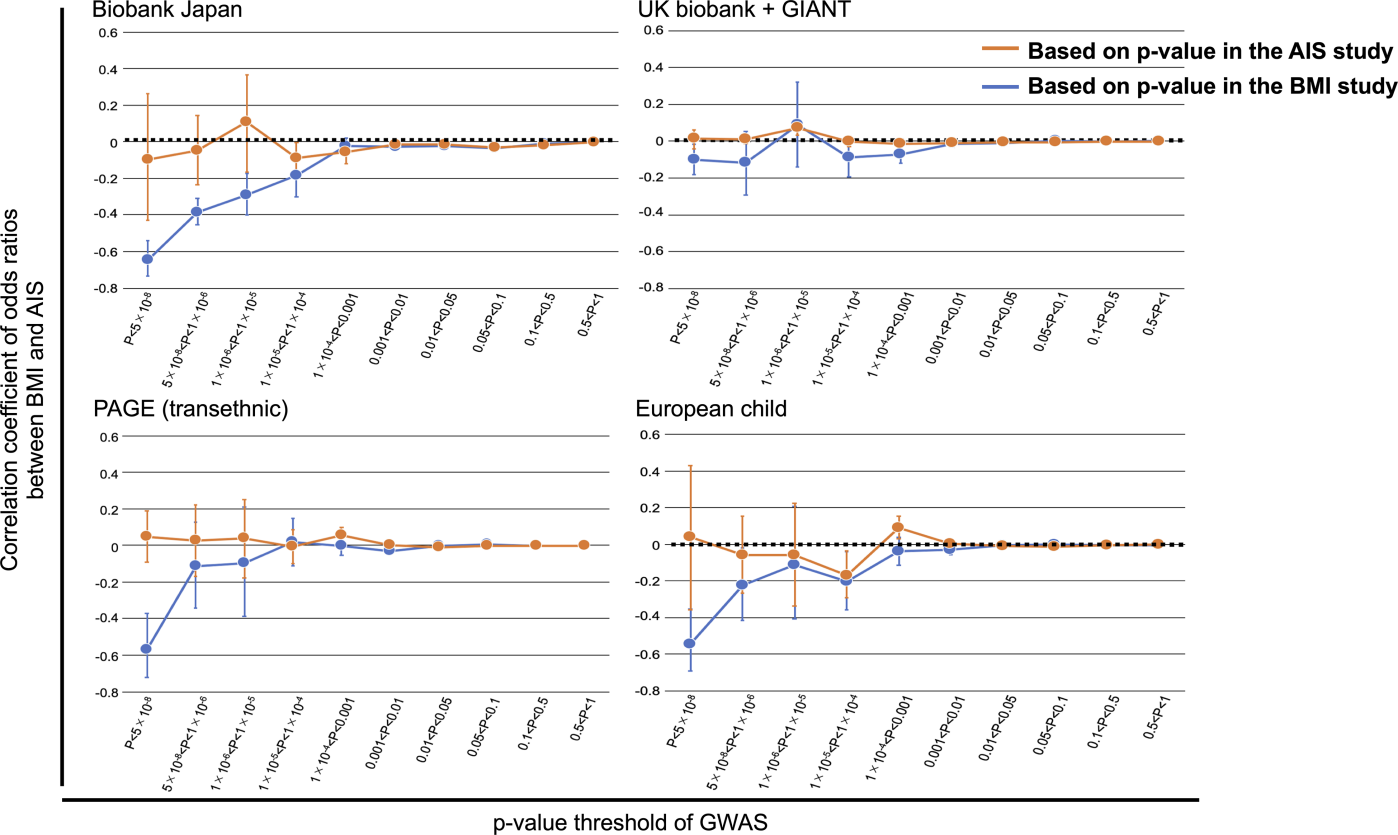
Overlap of the association with BMI and AIS. Correlations were evaluated for sets of SNPs stratified by the thresholds based on the *P* values of GWAS in each study after pruning the SNPs by LD (*r*2 < 0.3). For genome-wide SNPs, BMI or AIS susceptibility loci were excluded. The correlation coefficient and 95% CI are shown on the y-axis. Significant negative correlations of odds ratios for the SNPs related to BMI were observed, even for those showing modest association (P<0.0001 in the GWAS for BMI of BBJ). Similar trends of correlation were also observed using the BMI summary statistics of PAGE and European children. On the other hand, no modest correlations for the SNPs in p*-*value based on GWAS for AIS. GIANT, Genetic Investigation of Anthropometric Traits; PAGE, Population Architecture using Genomics and Epidemiology.

We further tried to provide evidence to support the causal relationship between low BMI on the development of AIS (and not vice versa). We conducted polygenic assessment for common variants shared between GWAS summary statistics for AIS and BMI to compare genetic backgrounds between BMI and AIS. We stratified the SNPs based on the p-values of GWAS (P) in each summary statistic and evaluated the correlation of odds ratios of the SNPs between the two traits. We observed significant negative correlations of odds ratios for the SNPs related to BMI, even for those showing modest association (P<0.0001 in the GWAS for BMI of Biobank Japan; *r*=-0.65~-0.18 for each *P* value range, p-value<0.005 for each correlation test) (**Figure 3**), confirming agreement in direction of genetic risk in a polygenic manner (40, 41). Observed correlations (r) of odds ratios indicate substantial overlap of the genetic risk (with opposing directions of effects) between the two traits, not only in the identified BMI susceptibility loci exceeding the GWAS significant level but also at the loci showing nonsignificant associations. These findings are consistent with the negative relationship between AIS with BMI reported in the previous studies (12, 16). When repeating these analyses using other BMI summary statistics from other populations, similar trends of correlation of odds ratios were observed in the analyses using the BMI summary statistics of transethnic analysis (PAGE) and European child. On the other hand, we did not observe modest correlations of odds ratios for the SNPs in p*-*value based on GWAS for AIS (**Figure 3**).

## Discussion

Previous epidemiological studies have reported an association low BMI with AIS (14, 15) in several populations. However, the causality has been proven by the previous epidemiological studies because BMI may be correlated with other factors. MR is a method based on genetic study and estimates causal inferences between traits. And one of the characteristics of genetic studies is basically independent of the environment, which differentiates MR from epidemiological studies and is its advantage. Therefore, we conducted MR analyses using summary statistics from the largest GWAS meta-analyses of BMI and AIS. We found significant evidence of genetically low BMI having a causal effect on onset of AIS. Our results are consistent with those of epidemiological studies showing the correlation AIS with low BMI (our previous GWAS for AIS was only able to show a genetic correlation, considering polygenic effect based GWAS summary statistics, between AIS and BMI in Japanese). Therefore, this is the first study revealing the causality between the two in genetic aspect.

Our study also showed the direction of effect: the variants having decreasing effects on BMI could be risk of onset of AIS, but the risk variants of AIS would not be risk for low BMI. This finding is consistent with epidemiological knowledge in relation AIS with BMI (14).

Previous studies have reported that AIS patients generally have a low BMI, with 25% having BMI scores in the anorexia region (42, 43). Low BMI may indicate eating disorders, excessive exercise, bone loss, and hormonal imbalances. And all of these may be associated with AIS trough lowering BMI. However, it is important to note that the distribution of BMI in the Japanese population is different from that of Western populations. According to the previous study, the non-Asian population has a higher a higher BMI than Asians (44). Since the distribution of BMI varies by population, it would be important to treat BMI as a continuous variable and estimate the risk of developing AIS, rather than uniformly applying a BMI threshold to individuals with different ratios across the distribution.

Since the relation of AIS with low BMI has been observed in Japanese as well as other populations (15), we repeated MR analyses using another summary statistic of GWAS for AIS derived from US cohort (European ancestry) and using summary statistics of GWAS for BMI derived from mainly European ancestry. GWAS summary statistics showed a significant correlation between Japanese and Caucasian in AIS. This result indicates that the genetic architecture of the AIS has a large portion in common among these populations. Therefore, based on the hypothesis that causal variants are common, MR was performed for each population. Even though the power calculation for MR analyses showed low statistical power of the US AIS cohort due to the sample size, the consistent trends with those of Japanese GWAS for AIS were replicated. This finding indicates that low BMI is a common risk factor for onset of AIS could among Japanese and European ancestry. Since most of GWAS for BMI are based on the BMI after becoming adults, the best GWAS data for BMI to be used together with GWAS for AIS is the results based on BMI measured in adolescents. In order to address this issue, we performed MR analyses using summary statistics of GWAS for BMI based on European children’s data. Consequently, the results were consistent with those of using adult’s results. These indicate the variants affecting BMI are shared between adults and children, and strengthens the results using BMI summary statistics derived from adults.

The US GWAS results of AIS contained a small sample size which led to low statistical power and our Japanese AIS GWAS is the largest one which means summary statistics are more accurate. On the contrary, BMI GWASs has been mainly reported based on Caucasians. Since MR using summary statistics from different populations is not recommended due to differences in LD, we take polygenic assessment for shared common variants between BMI and AIS. In addition, since it has been reported that Japanese lead variants in AIS have similar odds ratios to those of Caucasians and East Asians (25), we consider that the effect of LD differences is small. To take BMI results in the European and apply them to AIS results in Japanese would decrease power to detect negative relationship of BMI on AIS. However, we observed similar trends in various summary statistics. This indicates that even in the conservative approach, we could observe supportive relationship between BMI on AIS. There are indeed differences in genetic structure in the population due to LD structure, however, we consider that applying the instrumental variable to different populations is a rather conservative approach considering LD structure.

To make these findings more robust, further study with larger sample size is needed. Although it is difficult to improve the results in the current situation, it is expected that the results can be sufficiently improved by continuing the research and increasing the sample size. On the other hand, the estimate of MR analysis using European population (UK biobank and GIANT) summary statistic of BMI was relatively higher in comparison with the other instruments of BMI. These results suggest the possibility of pleiotropy. This is particularly remarkable in the European data and may reflect the results of epidemiological studies showing a positive correlation between AIS and BMI (45).

We conducted reverse MR to investigate the effect of AIS on the risk of low BMI. While less independent SNPs used for analyses compared to those of GWASs for BMI, the consistent results that the estimate of AIS instruments was around 0 in contrast to those of MR analyses were observed. These findings support that the risk variants of AIS would not be a risk of low BMI. And this study shows that genetically low BMI is a risk for onset of AIS, which suggests that children with low BMI should be screened for scoliosis at an early stage to potentiate early detection and intervention of AIS.

The Scoliosis Study Group recommends that all children between the ages of 10 and 14 have an examination for scoliosis once a year (46). This is because early scoliosis screening in children is beneficial for the early detection and prevention of spinal deformities and is associated with a better prognosis (46). Several developed nations, including the United States, Japan, and some European countries, have recognized the importance of scoliosis screening in children and have implemented scoliosis screening at the national level. With screening, these nations try to prevent and proactively manage spinal deformities and scoliosis in children (47–49). It is important to use various information, including epidemiology and genetic background, to detect scoliosis in its early stages. Further research is also needed to discover whether or not growing weight through diet can help prevent the onset and progression of scoliosis.

There are some limitations in this study. In a previous study, no major differences in genetic architecture between rare and common variants have been observed in Japanese AIS (12). Therefore, we do not consider it problematic to evaluate only the common variant for our study. However, the rare variant has not been evaluated among Caucasians. Future study is needed to confirm whether the trend is similar to that of the Japanese. Since sample size and number of significant SNPs of GWAS for AIS has been much smaller in comparison with GWAS for BMI. Unfortunately, we could not conduct reverse-MR analyses using AIS summary statistic of U.S cohort due to lack of significant SNPs. Recruiting samples and further studies are needed to deny the reverse causation (AIS on BMI) more convincingly and to develop insight of AIS. Especially, as our GWAS results are based on Asian population, replication using other population’s GWAS results are favorable. Since AIS is a disease caused by multiple factors, it is important to recognize that low BMI is one of the factors affecting the onset of AIS. And the identification of other factors involved in the onset of AIS is also necessary. While the results of MR-Egger intercept test and MR-PRESSO did not show any obvious pleiotropy, the possibility of pleiotropy cannot be completely ruled out at this time. Because previous studies reported the association of onset of AIS with low bone mineral density (BMD) (50) or low muscle mass (51). Considering the results of weighted median method, which use half of variants for analyses, there could be a mixture of variants in BMI associated SNPs that affect BMI directly and variants that affect BMI through other pathways. Further studies are demanded to clarify the variants which directly affect BMI. Then, we could evaluate the causality more precisely.

## Conclusions

This is a first study conducting MR analysis between AIS and BMI. We showed the possibility that genetically low BMI has a causal effect on onset of AIS. This result was consistent with those of epidemiological study and would contribute the early detection of AIS.

## Scottish Rite Hospital Clinical Group (SRHCG)

Lori A. Karol^1^, Karl E. Rathjen^1^, Daniel J. Sucato^1^, John G. Birch^1^, Charles E. Johnston III^1^, B. Stephens Richards^1^, Brandon A. Ramo^1^, Amy L. McIntosh^1^, John A. Herring^1^, Todd A. Milbrandt^2^, Vishwas R. Talwakar^3^, Henry J. Iwinski^3^, Ryan D. Muchow^3^, J. Channing Tassone^4^, X. -C. Liu^4^, Richard Shindell^5^, William Schrader^6^, Craig Eberson^7^, Anthony Lapinsky^8^, Randall Loder^9^ and Joseph Davey^10^

1. Department of Orthopaedic Surgery, Texas Scottish Rite Hospital for Children, Dallas, Texas, USA.

2. Department of Orthopaedic Surgery, Mayo Clinic, Rochester, Minnesota, US

3. Department of Orthopaedic Surgery, Shriners Hospitals for Children, Lexington, Kentucky, USA.

4. Department of Orthopaedic Surgery, Children’s Hospital of Wisconsin, Milwaukee, Wisconsin, USA.

5. OrthoArizona, Phoenix, Arizona, USA.

6. Departments of Orthopaedics, Sports Medicine, and Surgical Services, Akron Children’s Hospital, Akron, Ohio, USA.

7. Pediatric Orthopaedics and Scoliosis, Hasbro Children’s Hospital, Providence, Rhode Island, USA.

8. University of Massachusetts Memorial Medical Center, Worcester, Massachusetts, USA.

9. Indiana University-Purdue University Indianapolis, Indianapolis, Indiana, USA.

10. University of Oklahoma Health Sciences Center, Oklahoma City, Oklahoma, USA.

## Data availability statement

Publicly available datasets were analyzed in this study. This data can be found here: http://jenger.riken.jp/.

## Ethics statement

All genomic DNA from patients were examined after obtaining informed consent. The Medical Ethics Committee of the Keio university hospital, Tokyo, approved the study protocol (20080129). We have obtained written informed consent for publication of clinical details of the patients.

## Author contributions

CT, SI, KW, and CW supervised the project. NO and CT designed the project and provided overall project management. NO and CT drafted the manuscript. YM and MiK performed the genotyping for the GWAS. CT, NO, YK, Yuk, MaK and AK analyzed the GWAS data and performed integrative analyses. NO, KT, YO, YoT, SM, NK, KU, MI, TaS, KeW, TaK, HY, HT, KH, YuT, ToK, TT, TS, HSh, AI, SaI, TS, NF, MY, MN, KC, KaK, TsS, TA, KN, KeK, HSu, TI, SD, RS, NH, EO, MM, KW, and JH collected and managed clinical data. All authors contributed to the article and approved the submitted version.
